# Challenges and opportunities in achieving effective regulatory T cell therapy in autoimmune liver disease

**DOI:** 10.1007/s00281-022-00940-w

**Published:** 2022-05-31

**Authors:** N. Richardson, G. E. Wootton, A. G. Bozward, Y. H. Oo

**Affiliations:** 1 Centre for Liver and Gastrointestinal Research & NIHR Birmingham Liver Biomedical Research Unit, Institute of Biomedical Research, Institute of Immunology and Immunotherapy, University of Birmingham, Birmingham, B15 2TT UK; 2 Advanced Cellular Therapy Facility, University of Birmingham, Birmingham, B15 2TT UK; 3 Centre for Rare Diseases, European Reference Network Rare Liver Centre, University Hospital Birmingham NHS Foundation Trust, Birmingham, B15 2TH UK; 4 Liver Transplant and Hepatobiliary Unit, Queen Elizabeth Hospital, University Hospital Birmingham NHS Foundation Trust, Birmingham, B15 2TH UK

**Keywords:** Regulatory T cell, Autoimmune liver, Cell therapy, Liver microenvironment

## Abstract

Autoimmune liver diseases (AILD) include autoimmune hepatitis (AIH), primary biliary cholangitis (PBC) and primary sclerosing cholangitis (PSC). These immune-mediated liver diseases involve a break down in peripheral self-tolerance with largely unknown aetiology. Regulatory T cells (Treg) are crucial in maintaining immunological tolerance. Hence, Treg immunotherapy is an attractive therapeutic option in AILD. Currently, AILD do not have a curative treatment option and patients take life-long immunosuppression or bile acids to control hepatic or biliary inflammation. Clinical investigations using good manufacturing practice (GMP) Treg in autoimmune liver disease have thus far demonstrated that Treg therapy is safe and that Treg migrate to inflamed liver tissue. For Treg immunotherapy to achieve efficacy in AILD, Treg must be retained within the liver and maintain their suppressive phenotype to dampen ongoing immune responses to hepatocytes and biliary epithelium. Therefore, therapeutic Treg subsets should be selected for tissue residency markers and maximal functionality. Optimisation of dosing regime and understanding longevity of Treg in vivo are critical to successful Treg therapy. It is also essential to consider combination therapy options to complement infused Treg, for instance low-dose interleukin-2 (IL-2) to support pre-existing and infused Treg survival and suppressive function. Understanding the hepatic microenvironment in both early- and late-stage AILD presents significant opportunity to better tailor Treg therapy in different patient groups. Modification of a hostile microenvironment to a more favourable one either prior to or during Treg therapy could enhance the efficacy and longevity of infused GMP-Treg. Applying recent technology to discovery of autoantigen responses in AILD, T cell receptor (TCR) sequencing and use of chimeric antigen receptor (CAR) technology represents the next frontier for disease-specific CAR-Treg therapies. Consideration of all these aspects in future trials and discovery research would position GMP Treg immunotherapy as a viable personalised-medicine treatment option for effective control of autoimmune liver diseases.

## Autoimmune liver diseases

Autoimmune hepatitis (AIH), primary biliary cholangitis (PBC) and primary sclerosing cholangitis (PSC) are immune-mediated liver diseases characterised by a loss of immunological tolerance to hepatocytes and biliary epithelial cells [1–3]. The triggering factor(s) for onset of AILD is still unknown. It is widely accepted that effector CD4 (Th1/Th17/T follicular helper) and CD8 T cell immune responses to self autoantigen(s) (expressed by hepatocyte and/or biliary epithelium) are major contributor(s) to the pathogenic mechanisms involved in AILD [4–8]. Humoral responses driven by plasma cells [9, 10] are then licensed to perpetuate long-term damage against liver tissues. Innate immune cells (NK and macrophages) are recruited to the site of liver insult and can exacerbate inflammatory pathways [11–14].

Damage to hepatocytes and biliary cells is counteracted by Treg in combination with other immune cells, including myeloid-derived suppressor cells [15, 16] and tolerogenic dendritic cells [17]. If initial insult is not controlled, this can lead to chronic active hepatitis/cholangitis which can be followed either by repair processes that accompany regeneration and/or by fibrosis. Over time ongoing fibrosis can lead to liver cirrhosis, liver failure and liver cancer [18].

Recent advances in technology have enabled us to study hepatic immune cells at the single-cell level [19, 20]. Further application in AILD would allow investigators to understand immunological behaviour in different autoimmune liver patient groups, map T cell receptor (TCR) and B cell receptor (BCR) repertoires and potentially uncover signatures which correlate with disease severity, activity and response to treatment.

## Regulatory T cells in AILD

Regulatory T cells (Treg) are essential for the active maintenance of peripheral tolerance. Both in humans and mice, CD4^+^CD25^high^ Treg constitute 5–10% of peripheral CD4 T cells in the blood, and they play a crucial role in maintaining immunologic self-tolerance by actively suppressing self-reactive lymphocytes [21, 22]. Depletion of CD4^+^CD25^high^ Treg in rodents results in spontaneous development of multiple organ-specific and systemic autoimmune diseases as well as promoting anti-tumour immunity, whilst reconstitution of Treg prevents autoimmune disease development [22–24]. Treg constitutively express the transcription factor FoxP3, a ‘master controller’ of their development and function [25, 26] and expression of IL-7 receptor (CD127) inversely correlates with Foxp3 in CD4^+^CD25^high +^ T cells [27, 28]. Therefore, Treg are currently defined as a subset of CD4 lymphocytes which are CD4^+^CD25^high^CD127^low^FoxP3^+^ [29].

The liver is a relatively immune-privileged site evolved to maintain a state of active tolerance to avoid unnecessary immune responses to food antigens and low levels of endotoxin from the gut via the portal vein, whilst maintaining the capacity to respond to infectious agents [30–32]. The liver is well adapted to maintain homeostasis due to its unique populations of antigen-presenting cells with tolerogenic characteristics, feedback mechanisms to control inflammation, high density of innate immune cells and a richness of suppressive soluble mediators [33]. However, in AILD, rampant inflammatory responses to self-antigen(s) are not adequately controlled by the liver’s tolerogenic mediators. As the liver is biased towards tolerance under normal conditions, it is hypothesised that external effects may be required to break tolerance in genetically predisposed individuals, potentially with weakened Treg control.

Whether Treg are reduced in frequency or functionality in AILD has been the focus in a number of studies, but due to difficulties in tissue collection and cell isolation, there is limited data available based on Treg isolated from autoimmune livers compared to non-autoimmune organ donors. Peripheral blood Treg frequency is therefore often used as a surrogate indicator of the liver setting. However, these studies do not have standardised design or comparable patient groups—therefore much of the data has been contradictory.

In AIH, some have reported significant defects in the Treg population, whilst others have reported that Treg are maintained [34–36]. Interestingly, patients with active AIH display increased Treg frequency both in the periphery and in liver tissue sections [36, 37], suggesting that quality of Treg may be more relevant than frequency alone to disease control. Most importantly, Treg isolated from autoimmune hepatitis liver are still functional [38].

The frequency of Treg in peripheral blood of PSC patients and both peripheral blood and liver of PBC patients has been found to be significantly decreased [39, 40], suggesting that Treg insufficiencies in cholangitis may play a role in disease pathology.

In vitro models have shown that Treg isolated from autoimmune livers are functionally suppressive after having migrated via the transendothelial route across a cholangiocyte layer [38]. Therefore, it is thought that by enriching for functional Treg in the liver, the balance between effector T cells and Treg may be able to be restored and disease severity moderated.

At present, autologous GMP Treg therapy using isolated peripheral Treg expanded ex vivo has been shown to be safe in a wide range of autoimmune and transplantation settings, including in the liver [41–44]. To deliver a successful and efficacious Treg therapy to treat autoimmune liver diseases, there are multiple challenges and opportunities which should be taken into account and we will discuss in this article.

## Key challenges and opportunities

### Liver homing, retention and survival of regulatory T cells

**Fig. 1:**
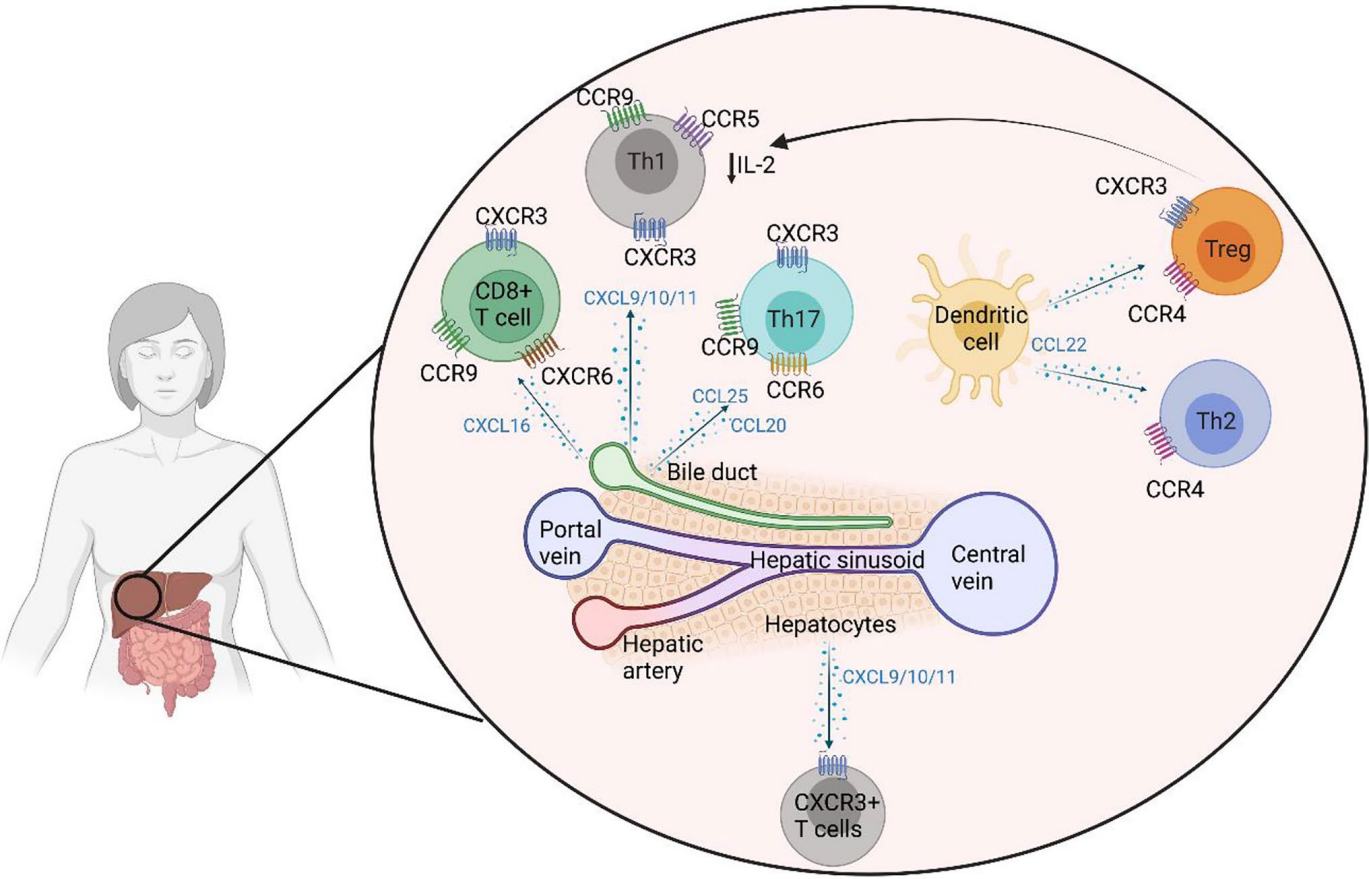
T lymphocyte migration, position and survival in human liver. *Hepatic* *T cells consist of effector CD8 T cells and CD4 T helper cells (Th). These cells are constantly controlled by regulatory T cells (Treg) to maintain hepatic tolerance. Both effector T cells and regulatory T cells are recruited by chemokine receptor, CXCR3. Chemokine CXCL9, 10 and 11 are ligands for CXCR3 and these ligands are expressed on inflamed hepatic sinusoids. In the context of inflammatory bowel diseases, gut homing lymphocytes (mainly Th17 and CD8 cells) which express CCR9 are recruited to PSC liver *via* chemokine CCL25 which is the ligand for CCR9 receptor and is expressed on inflamed hepatic sinusoidal endothelium. Thus, the CXCR3-CXCL9-11 pathway is crucial for T cell recruitment to inflamed AIH and PBC livers and CCR9-CCL25 axis is essential of gut homing lymphocyte migration to PSC livers. Once T cells are recruited, their positioning around hepatic parenchyma cells (hepatocytes), epithelial cells (biliary epithelium) and professional antigen-presenting dendritic cells also depends on chemokines. Both inflamed hepatocytes and biliary epithelium secrete CXCL9, 10 and 11 chemokines which position CXCR3*^+^ *CD8 T cells, Th1, Th17 and Treg to localise around inflamed hepatocytes and biliary epithelium. Inflamed biliary epithelium secretes CCL20 and attracts CCR6 expressing Th17 cells. Intrahepatic dendritic cells secrete CCL22 thus CCR4 expressing Treg reside in close proximity to exert their suppressive function together. Hepatic effector T cells (CD8, Th1 and Th17) secrete IL-2, which is crucial not only for autocrine survival of these effector T cells but also for Treg which highly express IL-2 receptor, CD25. Intrahepatic IL-2 level has been reported to be minimal due to continuous consumption of IL-2 pool by immune cells including Treg for their survival*

Chemokines are secreted by hepatocytes, bile ducts and stromal cells in human liver and act like sign-posts for immune cell migration and tissue infiltration (Fig. 1). Treg migration and infiltration into the liver tissue could be achieved with greater specificity by taking advantage of these chemokine-receptor interactions.

#### CXCR3

The chemokine receptor CXCR3 is expressed at high levels on Treg derived from human diseased liver whereas healthy liver Treg express significantly lower levels CXCR3 [45]. CXCR3 is required for stable adhesion to human endothelial sinusoidal cells (HSEC) via the chemokines CXCL9-11, which are only produced at detectable levels in diseased livers [46]. HSEC further upregulate these chemokines in vitro when under stress and in response to inflammatory signals [46]. Stable contacts between HSEC and lymphocytes subsequently lead to activation of integrins LFA-1 and VLA-4 on the lymphocyte surface which interact with cell adhesion molecules (ICAM, VCAM and VAP-1) expressed by inflamed HSEC which in turn allow transendothelial migration of Treg into the liver tissue [47]. Blocking CXCR3 inhibits Treg migration across the endothelium which suggests the significance of CXCR3 as a crucial liver homing marker for Treg. This evidence leads us to conduct an early-phase, proof-of-concept GMP Treg homing trial in autoimmune liver diseases (**AUTUMN**: **A**utologous reg**U**latory **T** cells inf**U**sion and tracking in autoi**M**mu**N**e hepatitis), tracking infused Treg over the first 72 h [48]. Over 80% of the AIH Treg expressed CXCR3 and between 22 and 44% of GMP Treg migrated to the site of liver inflammation [48].

#### CCR4

Chemokines CCL17 and CCL22 are secreted by liver dendritic cells (DC) at increased levels in chronically inflamed livers, recruiting Treg to the site of inflammation [45]. Upon Treg-DC interaction, Treg inhibit DC maturation and subsequently prevent T effector stimulation. Therefore, CCR4^+^ Treg may also be more likely to home to autoimmune liver and contribute to re-establishment of hepatic immune homeostasis.

#### CCR6

In PBC and PSC, inflamed bile ducts express chemokine CCL20. Biliary epithelial cells also secrete CCL20 in the presence of inflammatory cytokines (IL1β, TNFα, IFNγ and IL-17) resulting in the recruitment of CCR6 expressing Th17 T effector cells [49, 50]. Treg phenotypically mirroring Th17 also express CCR6 and IL-17 [51], but can readily convert to pathogenic Th17 in the inflamed environment [52]. As such, therapeutic infusion of large numbers of CCR6^+^ Treg should be avoided in the context of PSC/IBD and PBC where disease activity is already skewed towards Th17 [20, 50, 53].

#### CCL25

In normal conditions, CCL25 chemokine is expressed by gut epithelia and mucosa where it interacts with chemokine receptor CCR9 expressed on the surface of lymphocytes. It has been investigated in the context of PSC due to the strong association of this form of AILD with inflammatory bowel diseases (Crohn’s disease and ulcerative colitis). PSC liver has been found to express high levels of CCL25, produced by a range of cell types: portal DCs, macrophages and HSEC. This CCL25 appears to be specific to PSC as patients with other AILD including PBC do not overexpress this chemokine [54, 55]. PSC liver-infiltrating lymphocytes express functional CCR9 suggesting that they originated in the gut mucosa and have later been recruited to the liver [5]. Blocking the interaction between CCL25 and CCR9 has the potential to reduce liver immune infiltrate in PSC.

#### IL-2

IL-2 is a growth factor cytokine required for Treg proliferation, survival and function via its heterotrimeric receptor (CD25/CD122/CD132) [56, 57]. Treg express the highest level of the high-affinity IL-2 receptor, CD25 of all immune cells and are therefore highly sensitive to extremely low amounts of IL-2. Intrahepatic Treg are no different, as without sufficient IL-2, these cells are more susceptible to Fas-mediated apoptosis [38]. However, the pro-inflammatory liver environment has little IL-2 available, making survival and proliferation difficult for Treg [38]. Since Treg do not produce their own IL-2, it is likely that they receive IL-2 signals produced by conventional activated T cells in the environment (Fig. 1). Low doses of exogenous IL-2 in vitro and in vivo have been shown to selectively expand Treg [58–61], making it an attractive option to expand and support these cells in autoimmune disease. Liver-derived Treg provided with low-dose IL-2 upregulate expression of Treg functional molecule, CTLA-4 via activation of the JAK3-STAT5 signalling pathway [61].

Low-dose IL-2 therapy aims to promote Treg survival and function without providing enough IL-2 to stimulate conventional T cells and other immune cells expressing IL-2R (e.g. NK cells, monocytes, Kupffer cells). There are multiple options regarding low-dose IL-2 therapy: (a) medicinal grade recombinant IL-2 cytokine, (b) IL-2 monoclonal antibody complexes, (c) IL-2 mutein selective for CD25.

Recombinant IL-2 (aldesleukin) has been utilised in a wide range of autoimmune conditions to date including autoimmune liver diseases AIH and PSC [59, 60, 62, 63]. Overall, low-dose IL-2 treatment (1–1.5 million IU/dose) has excellent safety profile and can selectively activate and expand Treg in vivo. Novel alternatives such as IL-2 mAb complex (IL-2-JES6-1) with increased affinity for CD25 have been shown to significantly increase numbers of Foxp3^+^Treg in murine models [64, 65]. Recently, the development of mutated forms of IL-2 with specific binding to CD25 (avoiding interactions with lower affinity IL-2R) has excited the field. To enhance IL-2 selectivity, mutations are introduced to reduce its CD122/CD132 affinity thus creating a CD25 dependency. This type of approach selectively expands Treg without significant effects on proinflammatory cytokine secretors: effector T cells and NK cells. The engineered IL-2 mutein-IgG complex supported selective Treg expansion over a wide dose range and resolved non-obese diabetes (NOD) in mice [66]. The promise of IL-2 mutein in human disease is now being realised after safety and dose finding studies supported the progression to a pioneering clinical trial of PT101 IL-2 mutein complex in patients with active ulcerative colitis [67, 68] (ClinicalTrials.gov NCT04924114).

Thus, selectively expanding Treg which express a favourable combination of tissue homing markers with the ability to respond to survival factors (either natural or delivered as a combination therapy) is a reasonable approach in future for successful Treg therapy in autoimmune patients.

### Selection of Treg for treatment of AILD

#### Naïve vs memory

Administration of high purity, lineage stable and functionally effective Treg is fundamental to furthering tolerogenic cell therapy. Miyara and Sakaguchi first delineated human Treg into three subsets: naive/resting (CD45RA^+^FoxP3^low^), activated/effectors (CD45RA^−^FoxP3^high^) and cytokine-producing (CD45RA^−^FoxP3^low^) [69]. The application of mass cytometry and single-cell RNA sequencing has since revealed striking Treg heterogeneity, with Treg isolated from human PBMC subdivided into over 20 clusters including naïve, memory, Th1-like, Th2-like, Th17-like and Tfh-like Treg [51, 70]. Hence, it is possible to cell sort for a specialised Treg population either before or after in vitro expansion to maximise the effectiveness of the cell therapy.

Naïve CD45RA^+^ Treg expand readily in vitro, generating a homogenous Treg population without significant loss of FoxP3 expression or suppressive function [71]. During the expansion process, naïve Treg lose their CD45RA expression and gain CD45RO and CD69 which are useful tissue residency markers [72]. Conversely, central and effector memory Treg require addition of mammalian target of rapamycin (mTOR) inhibitor rapamycin to stabilise their FoxP3 locus and Treg lineage stability [73]. Many trials therefore focus on the selection of CD45RA^+^-naïve Treg prior to expansion and therapeutic infusion [53, 74].

In older adults as well as in chronic autoimmune diseases, there are significantly reduced naïve Treg in circulation, presumably as many have become antigen-experienced during their lifetime, which makes selection of these cells prior to therapeutically relevant expansion challenging.

#### Natural vs induced Treg

In the vast majority of pre-clinical and clinical investigations of Treg adoptive cell therapy, natural Treg (CD4^+^CD25^high^CD127^low^FoxP3^+^) cells are isolated directly from peripheral blood prior to expansion. Induced Treg (iTreg) can be generated in vitro by antigenic stimulation/CD3 activation of conventional T cells in the presence of TFG-β and IL-2. iTreg are typically functionally unstable and easily revert back to Th cell phenotypes. By assessing the Treg-specific demethylation region (TSDR) pattern of the FoxP3 locus, iTreg can be distinguished from naturally occurring thymic or peripheral Treg. The more demethylated the TSDR, the more epigenetically and functionally stable the cell (thymic Treg > peripheral CD45RA^+^ Treg > peripheral CD45RA^−^ Treg > iTreg).

However, recent developments from the Sakaguchi laboratory have showcased novel methods for generating stable iTreg with similar suppressive potential and TSDR to natural Treg. They have described use of cell cyclin-dependent kinase CDK8/19 inhibition to effectively convert antigen-specific conventional T cells into induced FoxP3 + Treg with the capacity to ameliorate disease activity in murine autoimmune diabetes and encephalomyelitis [75]. These CDK8/19 blockade-induced Treg were not fully demethylated at the FoxP3 TSDR; therefore, further screening revealed the importance of CD28-PKC-NF-kB signalling to FoxP3 demethylation. Blockade of CD28 signalling in effector T cells promoted the generation of induced Treg with similar TSDR to natural Treg [76]. These CD28-deprived iTreg were highly suppressive in an antigen-specific manner in vivo, prevented T effector IFNγ production and retained their lineage stability even after residence in inflamed skin tissue. As such, inhibition of CDK8/19 and/or CD28 signalling blockade represent novel means to achieve large numbers of functional, stable iTreg from disease-mediating T conventional for human therapeutic applications. Using such a method would circumvent the need to isolate rare Treg and the lengthy Treg expansion protocols (typically 4–6 weeks) required to produce therapeutically relevant cell numbers.

#### CTLA-4

Cell-contact cytotoxic T lymphocyte antigen-4 (CTLA-4)–dependent suppression plays an important role in regulatory T cell function by competition for the co-receptor CD28 [77] and active removal of co-stimulatory ligands CD80/86 from the surface of antigen-presenting cells, rendering them unable to sufficiently stimulate nearby T cells [78]. Our previous work suggests that cell surface CTLA-4 is selectively upregulated on Treg when exposed to low-dose IL-2 in vitro [79]. Low-dose IL-2 resulted in selective activation of STAT5 by phosphorylation whilst effector T cells and NK cells exposed to the same dose were not activated. STAT5 signalling in AIH and healthy donor PBMC as well as AILD hepatic infiltrating lymphocytes leads to increased CTLA-4 expression and therefore greater suppressive potential^78^.

#### Hepatic tissue homing and localisation markers

As discussed previously, tailoring the Treg subset(s) used in cell therapy to the tissue homing chemokine receptors relevant in disease of interest can promote infused GMP Treg reaching the target tissue. In the case of AILD this could include Treg expression of CXCR3 to facilitate recruitment to inflamed autoimmune livers, CCR6 and CXCR3 to position Treg around inflamed hepatocytes and biliary epithelium and CCR4 for close localisation of Treg around DC to enhance Treg suppressive function, survival and proliferation.

There is a concern that certain autoantigens present in AILD, particularly in AIH type 1, are not liver-specific and that this could in turn influence the effectiveness of Treg therapy. However, early evidence from the AUTUMN trial showed that a high proportion of infused autologous Treg cells expressed CXCR3 and that 22–44% of homed to the liver in AIH type 1 patients. The remainder of the cells were identified within the spleen and bone marrow, and no Treg cells were detected in the lungs or brain. This along with very good safety profiles from completed liver transplantation Treg infusion trials helps build confidence that therapeutic Treg from autoimmune liver patients are most likely to home to the liver as the site of the inflammation [80–82]. Similar whole-body imaging studies would be sensible in comorbid patients with AILD and extra-hepatic inflammatory/autoimmune conditions prior to Treg therapy application, to ensure that Treg distribute to disease site(s) effectively and do not get ‘trapped’ in off-target tissues. Such off-target effects may lead to over-suppression of immune responses in non-target organs, rendering the patient more vulnerable to infection or over the longer term, development of cancers. As Treg therapy is in its infancy, there has not yet been sufficient time or numbers of treated patients to monitor the malignancy developing as a result of this treatment.

#### Metabolic functions

Treg typically express high levels of CD39, an ectonucleotidase which degrades ATP to release adenosine [83]. Adenosine interacts with A2aR receptors expressed on a range of innate and adaptive immune cells which suppresses secretion of neutrophil chemo-attractants to dampen the inflammatory response [84]. CD39^+^ Treg have been suggested to be more effective in suppression of T effector proliferation and cytokine production compared to their CD39^−^ Treg counterparts [85–87]. In AIH patients, CD39^+^ Treg are reduced in frequency, generate less adenosine and are more susceptible to conversion into pathogenic IFNγ or IL-17 producing cells when exposed to inflammatory stimuli compared to healthy age-matched controls [88]. Human Treg expressing CD39 also maintain FoxP3 expression more efficiently over a culture period than those with low CD39 expression. High CD39 expression is associated with high CD25 and CTLA-4 expression alongside low levels of CD127 [86]. As such, CD39 may be a relevant marker for selection of optimised GMP-Treg in future works.

Recently, AMP-activated protein kinase alpha 1 (AMPKα1) has been shown to be important in maintaining the immunosuppressive function of Treg in murine AILD. Specific knockout of Treg AMPKα1 led to significant lymphocyte infiltration in the liver and subsequent liver injury. AMPK-deficient Treg did not inhibit proliferation of T effectors as efficiently as in WT mice [89]. Importantly, differential activity of AMPK has also been identified in PBC patients, with AMPK inhibition now representing a new avenue to support Treg function in PBC.

### Inflamed liver microenvironment shapes Treg stability and function

Inflamed intrahepatic microenvironment is enriched for pro-inflammatory cytokines, including IL-6, IL-12, IFNγ and TNFα [38]. They play a crucial role in recruitment, differentiation, survival and proliferation of immune cells within the autoimmune tissue. Exposure to predominantly pro-inflammatory signals can lead to a reduction in the potency of Treg cells or resistance of T effector cells to Treg cell suppression [90]. In this pro-inflammatory setting, instability of the FoxP3 epigenetic signature leads to lineage plasticity and conversion of Treg into Treg/Th1 or Treg/Th17 hybrid phenotypes associated with pathogenicity and reduced function [91]. For example, the frequency of Th1-like Treg is increased in patients with autoimmune hepatitis [92]. Similarly, Th17-like Treg have been shown to be increased in psoriasis [93] and inflammatory bowel diseases [94]. Understanding the pressures faced by Treg when entering the inflamed liver and the impacts on Treg function and stability is vital for their therapeutic potential in liver diseases.

Aiming to moderate the hostile autoimmune liver environment to a more favourable one could take the form of IL-2, Treg survival and functional cytokine supplementation as described previously, or the use of monoclonal antibodies to block specific pro-inflammatory cytokine pathways. Such trials would be most informative in AILD, particularly in cases of recurrent AIH flares or PSC for which current treatment options are limited.

There are also questions to be addressed regarding metabolites and microbial peptides from the portal vein which can have an impact on metabolism, phenotype and function of intrahepatic Treg cells. Short-chain fatty acids (SCFAs) which are generated by bacterial fermentation of dietary fibre promote expansion of Treg. Low concentrations of butyrate facilitate differentiation of Treg in vitro and in vivo under steady-state condition but higher concentrations of butyrate inhibit histone deacetylase activity and therefore induced potentially pathogenic Th1 cell types (CD4^+^Tbet^+^IFNγ producers) [95]. Thus, SCFAs promote T cell differentiation into both effector and regulatory T cells to promote either immunity or immune tolerance depending on immunological context [96]. These factors all contribute to a highly complex setting which will determine the biology of GMP Treg cells once within the inflamed liver.

### Optimisation and frequency of dosing regime

Optimal dose and timing of polyclonal GMP Treg infusion is largely unknown as the field is still in its infancy and there is no internationally agreed set of standards related to the treatment. Data generated in type 1 diabetes has shown that delivery of up to 256 million Treg in a single dose was safe and had indications of disease-modifying effects including improvement in c-peptide level [41]. Their longitudinal tracking studies up to 12 months using stable deuterium labelling showed transferred Treg were relatively long-lived, with up to 25% of the peak level remaining in the circulation at 1 year after transfer [41]. In the diabetes patients treated, conventional immunosuppression was not required; therefore, this data is an excellent example of Treg longevity in an otherwise unmanipulated immune system. In the setting of renal and liver transplantation tolerance, polyclonal GMP Treg dose of up to 4 million cells/kg was shown to be safe [82] and has led to the ThRIL and ONE trials [81] (ClinicalTrials.gov: NCT02166177 and NCT02129881). Current data published from the ONE trial has shown promising outcomes for patients receiving 1 dose of polyclonal Treg a week after kidney transplantation; patients treated were more likely to be able to switch from dual/triple drug immunosuppression to stable low-dose monotherapy compared to the standard of care [97].

Recently the results from the first trial in which two infusions of polyclonal Treg was delivered in combination with IL-2 showed that the addition of low-dose IL-2 boosted Treg numbers and supported an increased proportion of infused Treg present post-90 days. Importantly, the detailed immune monitoring in this trial indicated that low-dose IL-2 delivered in their dosing strategy (0.33–1 million IU/day for 5 consecutive days) also increased activated NK, mucosal-associated invariant T and clonal CD8 + T cell populations, and therefore caution must be taken when delivering low-dose IL-2 therapy to avoid significant expansion of cytotoxic subsets. This off-target effect of low-dose IL-2 stresses the need to develop IL-2 therapy in different approach such as antibodies, engineered or even mutant IL-2 that can selectively expand Treg but not NK or CD8 + T cells.

Currently, dosing and timing of antigen-specific GMP Treg or CAR GMP Treg are in discovery science stage and there is no available clinical trial data on these more novel therapies.

### Targeting early autoimmune liver diseases

Observations of reduced Treg numbers and/or impairment of Treg function in AILD supports the application of autologous Treg cell therapy [34, 36, 39, 40]. In AILD with relapsing–remitting disease patterns (e.g. AIH, inflammatory PBC and AIH/PSC overlap), the timing of administration of autologous Treg to patients would be a critical factor to consider. It may be the case that Treg infused during remission have a better likelihood of lineage stability and efficacy in the absence of a highly activated, pro-inflammatory liver. For patients with ongoing active disease, it is likely that Treg therapy would need to be delivered alongside conventional immunosuppressants which is supportive of Treg biology and/or moderators of inflammatory cytokine signalling to engineer a more quiescent immune landscape. There is a general consensus in the field that application of Treg therapy is more attractive in early-stage liver disease before extensive fibrosis or liver cirrhosis sets in.

Normal liver and early disease of AILD (Child–Pugh A cirrhosis) has abundant hepatic stellate cells which form a hepatic stromal framework to support Treg proliferation. During chronic active hepatitis and cholangitis, immune cells lead to tissue apoptosis and necrosis. Subsequently, stellate cells participate in wound healing and initiate tissue repair pathways leading to fibrosis characterised by the accumulation of collagen and other extracellular matrix components. Thus, in the early stage of hepatic inflammation, hepatic stellate cells are abundant; however, these cells are replaced by pro-fibrosis myofibroblasts in later stage liver disease (Child–Pugh B and Child–Pugh C cirrhosis) [98]. The loss of hepatic stellate cells in late disease also prevents these cells from contributing to liver tolerance mechanisms and from producing retinoic acid which promotes generation of induced Foxp3 + regulatory T cells [99, 100].

In addition, the choice of immunosuppression in patients receiving Treg cell therapy requires consideration. In vitro, biological relevant doses of calcineurin inhibitors (tacrolimus, cyclosporine) and second-line therapies (mycophenolate mofetil and cyclophosphamide) induce apoptosis of both resting and activated Treg [101–104]. In contrast, rapamycin can selectively enhance Treg expansion and prevent the outgrowth of Th1 and Th17 cells [73].

### Isolation and expansion of Treg

Treg have been isolated according to GMP principles for use in clinical trials of autoimmune disease and transplant rejection from peripheral blood and umbilical cord blood, thus far using magnetic isolation approaches consisting of typically a depletion step to remove CD8 + and/CD19 + cells prior to enrichment for CD25 + cells [81, 105, 106]. This method yields Treg of approximately 80% purity—as depletion is not 100% effective and there is no removal of activated Tconv cells which express both CD127 and CD25. Therefore, expansion of these cells in vitro for therapeutic use leads to an impure product, with potential to have expanded significant numbers of pathogenic effector T cells. Trials have more recently focused on flow cytometry–sorted Treg cells to achieve CD4 + CD25 + CD127low Treg of 99% purity for expansion [41, 44]. A sorting-based platform also enables considerable flexibility in future to select the most optimal Treg subsets based on the patient group or disease features (CD45RA + naïve Treg or highly suppressive CTLA-4 + Treg as discussed previously).

GMP Treg expansion utilises anti-CD3/28 stimulation in the presence of IL-2 to support significant proliferation over 4–6 weeks. Additional agents including rapamycin, TGF-β and retinoic acid can help maintain a stable Treg phenotype during the course of expansion [107–109].

It is important to note that the reliance on complex Treg isolation protocols and lengthy expansion processes not only increases cost of delivery of Treg therapy, but also make its use for acute AILD presentation incredibly difficult. Based on current protocols, 4–6 weeks of Treg expansion makes this approach unsuitable for patients who are experiencing acute or rapidly progressing disease.

### Development of antigen/liver-specific Treg for treatment of AILD

Application of Treg therapy to autoimmune diseases has so far utilised polyclonal Treg with unknown antigen specificity; however, growing evidence from animal models indicates that antigen-specific Treg (Ag-Treg) cells may be more efficient in controlling pathological immune responses in a disease-specific manner. Ag-Treg should localise with improved selectivity to the tissue in which the cognate antigen is expressed at high levels, meaning that risk of off-target or systemic immune suppression is reduced. In the NOD model of diabetes, Ag-Treg possess much higher potency compared to polyclonal Treg, requiring 4–20 fold fewer cell numbers to achieve comparable or improved disease onset prevention [110–112]. Similar studies in EAE, the murine model of multiple sclerosis, showed that Ag-Treg effectively prevented disease where polyclonal Treg were insufficient [113]. At present, no clinical trials involving human Ag-Treg in autoimmune disease have been completed.

The improved disease-modifying potential and more defined localisation benefits of Ag-Treg are clear; however, generation of Ag-Treg in humans for therapeutic purposes remains incredibly difficult. To directly isolate tiny numbers of circulating Ag-Treg from peripheral blood relies on tetramers engineered to the T cell epitope of interest. Alternative approaches promote Ag-Treg during expansion by exposure of polyclonal Treg to the target antigen. Both approaches require considerable prior knowledge of the disease-associated antigens involved and understanding of how different patients at different stages of disease progression respond to autoantigens.

**Fig. 2:**
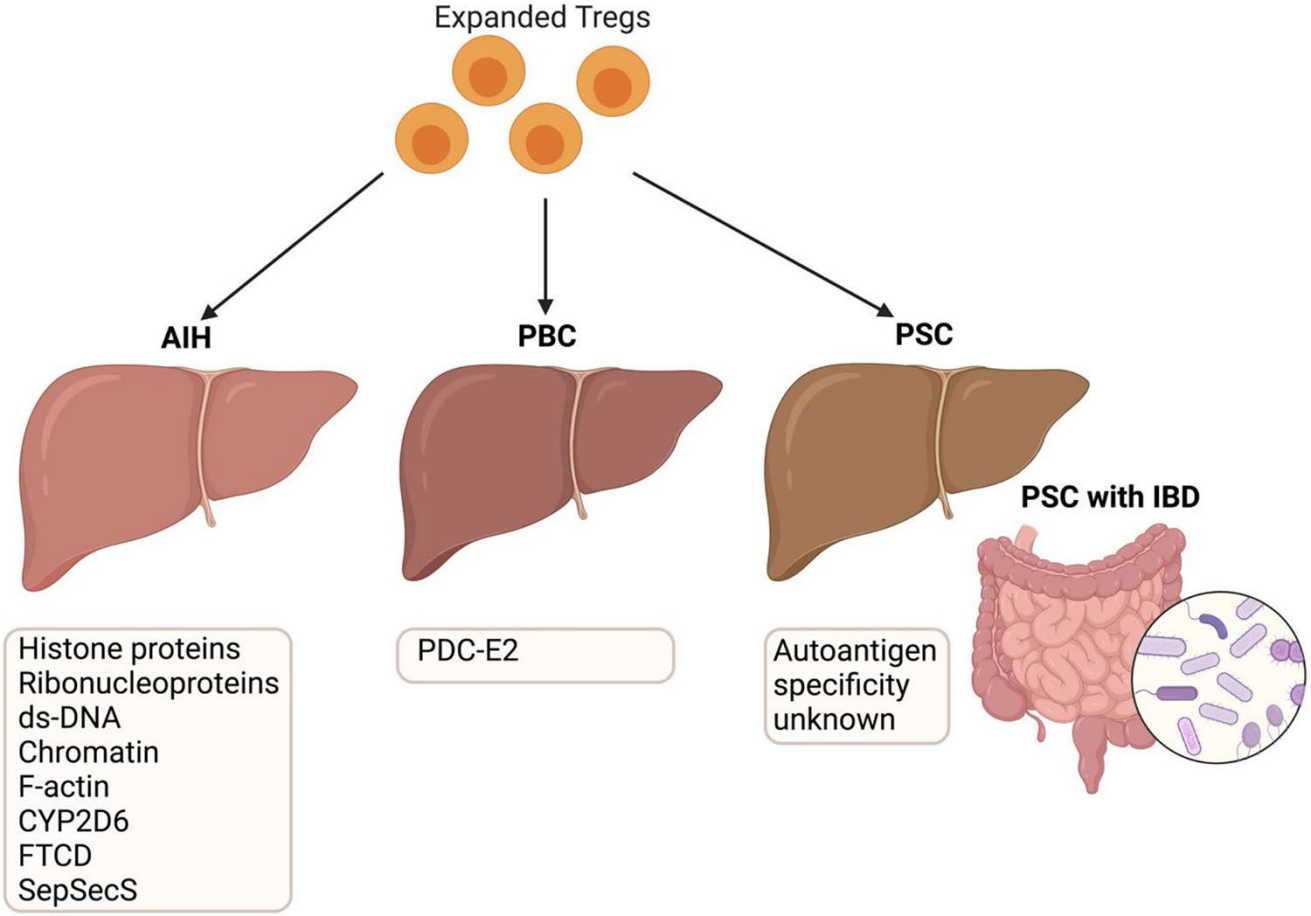
Antigens in different types of autoimmune liver diseases and their role in GMP Treg therapy. *There are known antigens in PBC; pyruvate dehydrogenase complex -E2 protein from biliary epithelium mitochondria (PDCE2) and type 2 AIH; cytochrome P450-2D6 (CYP2D6) and FTCD. AIH1 is associated with diverse antigens including histone proteins, ribonucleoproteins, double-stranded DNA, F-actin and SepSecS. Antigens involved in PSC are still unknown and may be liver or gut derived, considering that around 70% of PSC patients also have inflammatory bowel disease (IBD). Microbes and microbiome in inflamed small and large bowel have significant influence on IBD pathogenesis and disease progression and resolution. Clinical grade, good manufacturing practice (GMP) Treg is applicable in AIH, PBC and PSC to restore hepatic tolerance. Autologous Treg from AILD patient’s peripheral blood can be expanded in GMP cell culture media with cytokines and TCR stimulation to get suitable cell number for therapeutic infusion. GMP Treg could be applied as either polyclonal (type 1 AIH and PSC) or antigen-specific (type 2 AIH and PBC) in autoimmune liver diseases*

In AILD, many of the antigens that are targeted by autoantibodies have been identified (Table 1; Fig. 2). However, they are largely not disease or tissue restricted and it is unclear whether the presence of specific autoantibodies plays a significant prognostic role in determining the features of disease (likelihood of flares, treatment response etc.).

**Table 1:** Different types of autoimmune liver diseases and known autoantigen

Autoantibody	Disease relevance	Autoantigen(s) specificity	References
ANA (anti-nuclear antibodies)	AIH type 1	Histone proteins, ribonucleoproteins, ds-DNA and chromatin	[114–118]
SMA (anti-smooth muscle antibodies)	AIH type 1	F-actin	[119–121]
LKM-1 (liver-kidney microsome antibodies)	AIH type 2	Cytochrome p450 2D6 (CYP2D6)	[122–126]
LC-1 (liver cytosol-1 antibodies)	AIH type 2	Formiminotransferase cyclodeaminase (FTCD)	[127, 128]
SLA (soluble liver antigen antibodies)	AIH	SepSecS	[129–131]
AMA (anti-mitochondrial antibodies)	PBC	Pyruvate dehydrogenase complex subunit E2 (PDC-E2)	[132–134]

This table describes autoantibodies which are used in clinical application to identify the type of autoimmune liver diseases. It also mentions the currently known autoantigen for AIH type 2 and PBC. Autoantigen in type 1 AIH and PSC are still unknown.

Most autoantibodies detected in AIH (most notably ANA antibodies) are not disease-specific, being commonly expressed in systemic lupus erythematosus, rheumatoid arthritis and Sjogren’s syndrome, in addition to chronic viral liver disease patients (HCV, HBV). These autoantibodies are also not liver-specific, making targeting these responses more challenging due to the potential for off-target effects in multiple tissues. Further, 10–15% of patients do not have detectable autoantibodies at diagnosis using the current tests and are therefore categorised as ‘seronegative’ AIH.

PBC is characterised more simply by the presence of highly specific AMA (anti-mitochondrial antibodies) directed to the E2 subunit of pyruvate dehydrogenase complex (PDC-E2). More than 90% of PBC patients are AMA positive, making it a highly consistent autoantibody target used in diagnosis—although levels of AMA antibodies above the diagnostic threshold do not show prognostic value. Both early- and late-stage PBC patients have abnormal expression of the PDCE-2 antigen on the apical region of biliary epithelium, exposing this potent autoantigen to surveying T cells in the tissue. The triggering events leading to PDCE-2 exposure and ongoing T cell reactivity are unknown.

In contrast, PSC patients do not present with liver-specific autoantibodies, with more general cholestasis indicators (elevated serum alkaline phosphate levels) being the strongest biomarker available. Up to 80% of PSC patients are also comorbid for inflammatory bowel disease (IBD), making identification of liver-specific immune responses more challenging.

In settings where the disease-driving autoantigen(s) are poorly characterised (AIH type 1 and PSC), it may be sufficient to direct cells to the target organ rather than a specific antigen, as long as functional Treg accumulate within the inflamed tissue. This concept of ‘bystander suppression’ has been demonstrated in a number of mouse models of autoimmune disease and murine AILD—in which tolerance-inducing MHC-II carrying nanoparticles loaded with liver antigen CYP2D22 successfully induced antigen-specific tolerance to CYP2D22 and also prevented pathogenic immune responses to PBC-related liver antigen PDCE-2 [135]. If such a strategy were to be employed in human AILD, it would be essential to select suitable target antigens which were only localised within the target hepatic or biliary tissue to avoid systemic immune suppression.

To circumvent difficulties in isolation of antigen-specific Treg directly from patient starting material, efforts have moved towards in vitro generation of antigen-specific cells. This can be achieved by (1) expansion of polyclonal Treg in the presence of antigen-presenting cells primed with the target antigen, (2) induction of Treg from conventional T cells and (3) genetic editing of synthetic antigen receptors, i.e. TCR-Treg and CAR-Treg.

A lack of specific knowledge of disease-responsible antigen(s) and of the TCR sequences of T effector/memory cells that respond to these antigens represents a significant bottleneck in development of receptor-engineered Treg for the treatment of AILD. This information would enable the application of antigen-specific TCR-transduced Treg (TCR-Treg) or chimeric antigen receptor (CAR) technology to Treg therapies. TCR-Treg cells are ex vivo engineered regulatory T cells with inserted TCR relevant to the autoantigen and therefore can be generated in higher numbers than isolating Ag-Treg directly. CAR-Treg instead utilise a single-chain variable antibody fragment directed towards the target antigen fused to CD3 signalling domains and relevant costimulatory domains.

Advances in the field have already lead to the generation of alloantigen-specific CAR-Treg in human skin grafts [136] as well as autoantigen-specific CAR-Treg in mouse models of multiple sclerosis and anti-Factor VIII [137, 138]. Early-phase trials using HLA-A2 CAR-Treg in mismatch kidney transplantation and liver transplantation have started patient recruitment (ClinicalTrials.gov: NCT04817774 and NCT05234190).

Both TCR-Treg and CAR-Treg are manufactured initially in a similar way to polyclonal Treg: (1) GMP-grade isolation of natural Treg from peripheral blood mononuclear cells, (2) expansion of Treg using anti-CD3/CD28 stimulation to generate a ‘master’ Treg product. This polyclonal Treg product can then be transduced using a GMP-grade viral vector expressing the antigen-specific TCR/CAR construct. When manufacturing TCR/CAR-Treg, particular care must be taken to ensure a very high purity initial Treg isolation (> 95%) to avoid contamination of T conventional cells being transduced and therefore producing a product containing antigen-specific T conventional cells with disease exacerbating potential.

CAR-Treg can be further gene edited to improve the functionality of the cellular product—for example: overexpression of FoxP3, deletion of receptors which respond to inflammatory signals, insertion of tissue homing receptors or even insertion of ‘suicide gene’ cassettes which enable ablation of the infused cells to avoid long-term effects of the CAR-Treg product [139, 140].

## Conclusions

Adoptive cellular therapy exploiting regulatory T cells to treat autoimmune liver diseases is an attractive therapeutic option. The clinical aim for AILD is to control self-reactive effector T cells and reduce autoantibody levels and biochemical markers of liver damage (liver enzyme levels). However, the points raised in this article show several key areas where improvements to our understanding of Treg biology (localisation, frequency, function, plasticity, longevity and antigen specificity) both in non-autoimmune and autoimmune liver context are required. It is possible that combination of GMP Treg supplementation with other modalities of immune manipulation, including changing the inflamed liver microenvironment, may be required to achieve successful Treg therapy.

To develop suitable antigen-specific Treg therapy options (Ag-Treg, TCR-Treg and CAR-Treg) requires a more detailed picture of AILD patient responses to autoantigens. PBC and AIH type 2 have the most defined autoantigen profiles and are therefore most likely to be suitable candidates in which to develop liver Ag-Treg. Building upon CAR technology to engineer CAR-Treg targeting liver antigens with liver homing chemokine receptors and genetic switch to ablate infused cells would be a potential future option for AILD patients.

Crucial clinical and logistical challenges are also faced, including clinical trial design for timing of infusion, optimal dosing of Treg therapy, selection of appropriate patients with maximal potential to respond, cost and labour intensiveness of manufacturing and the multitude of combination therapy options which are available. Treg genomic and phenotypic analysis of autoimmune patients should also be incorporated to stratify and select the target patient cohort to apply GMP Treg therapy as personalised medicine. Initially it would be most sensible to apply Treg-based cellular therapies to lower-risk AILD patient groups who are stable on the current first-line drug treatments in order to monitor effects with reduced risk of severe disease progression. Treg therapy delivered to AIH patients in long-term remission could occur with removal of maintenance immunosuppression therapy; therefore, if patient liver inflammation begins to increase during the course of the trial, the patient can readily be diverted back onto the standard-of-care. However, higher-risk patient groups including PSC, in addition to AIH and PBC treatment non-responders, would benefit most from the development of novel therapeutic options.

With these challenges comes significant opportunity to develop improved therapies based on regulatory T cells and to improve the breadth of application to different diseases and different patient groups. A new era of personalised Treg therapy would potentially restore the immune tolerance in AILD patients and reduce the requirement for high-strength immunosuppressive drugs.

## Contribution to the field

This review summarises the overview and progress of Treg cellular therapy in autoimmune liver diseases. We also discuss the aspects requiring investigation for successful translation of Treg therapy in autoimmune liver diseases.
